# Necrobiosis lipoidica‐like lesions in a nondiabetic patient with systemic sarcoidosis: A case report and review of the literature

**DOI:** 10.1002/ccr3.3281

**Published:** 2020-08-30

**Authors:** Farnaz Araghi, Mohammadreza Tabary, Azadeh Rakhshan, Sahar Dadkhahfar, Reza M. Robati

**Affiliations:** ^1^ Skin Research Center Shahid Beheshti University of Medical Sciences Tehran Iran; ^2^ School of Medicine Tehran University of Medical Sciences Tehran Iran; ^3^ Department of Pathology Shohada‐e‐Tajrish Educational Hospital School of Medicine Shahid Beheshti University of Medical Sciences Tehran Iran; ^4^ Department of Dermatology Loghman Hakim Hospital Shahid Beheshti University of Medical Sciences Tehran Iran

**Keywords:** granulomatous disorders, necrobiosis lipoidica, sarcoidosis, skin pathology

## Abstract

Necrobiosis lipoidica‐like lesions, in known cases of sarcoidosis, can be considered as a member of the broad spectrum of histologic changes in sarcoidosis.

## Graphical abstract

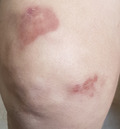

## 1 INTRODUCTION

Sarcoidosis and necrobiosis lipoidica (NL) are noninfectious granulomatous disorders with unknown etiologies.^1^ Sarcoidosis is a systemic disease with the hallmark of noncaseating granuloma formations. Red‐brown papule or plaques, erythema nodosum, and lupus pernio are the different cutaneous presentations of sarcoidosis.^2^ Necrobiosis lipoidica (NL) is a less common noninfectious granulomatous skin disease which is characterized by yellow‐brown, atrophic, telangiectatic plaques with an elevated violaceous rim, typically in the pretibial region ^3^ with a possible association with diabetes mellitus.^4^ Up to now, there have been some reports that revealed the association of sarcoidosis and NL, despite their distinctive histologic and clinical features.^1^, ^2^, ^5^, ^6^, ^7^, ^8^, ^9^ This report also deals with a known case of systemic sarcoidosis that developed necrobiosis lipoidica‐like lesions on the leg.

## 2 CASE REPORT

**FIGURE 1:**
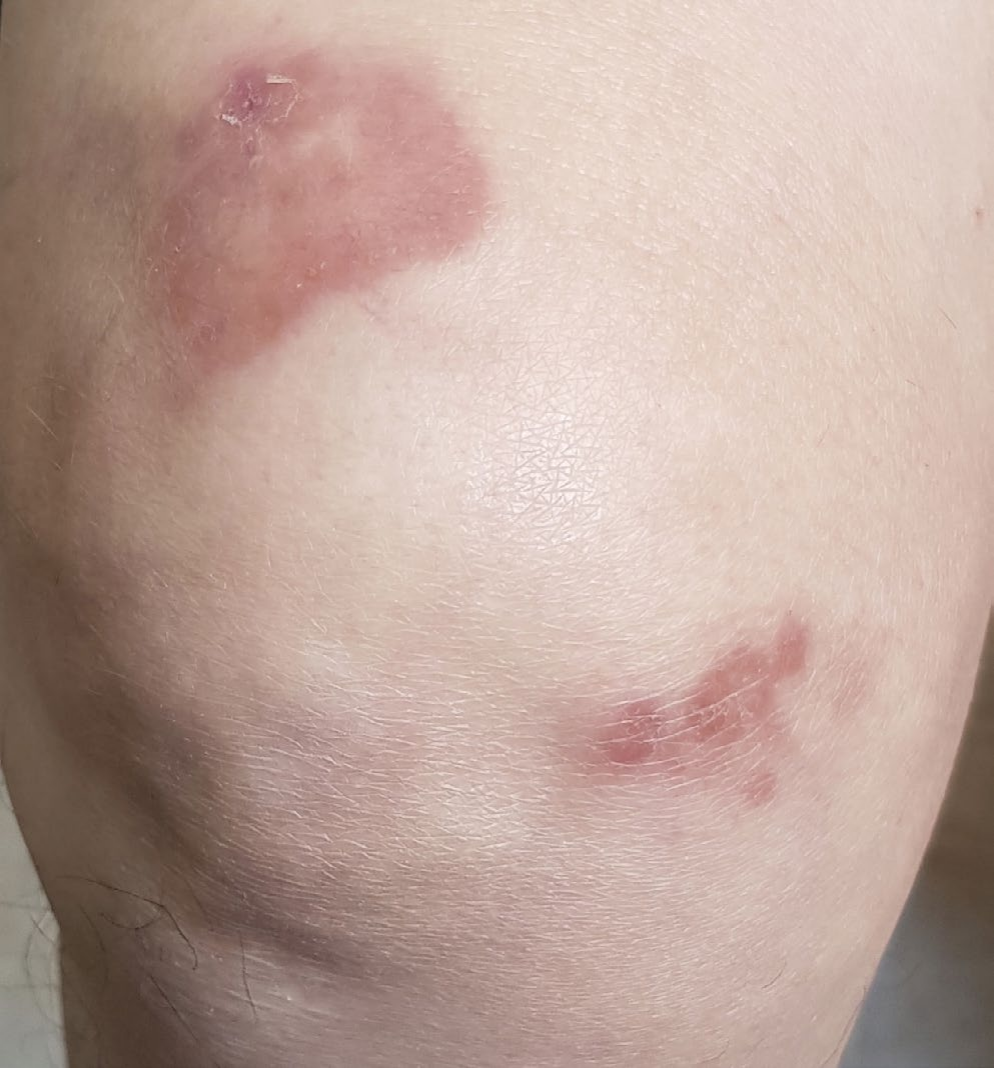
Erythematous yellow‐red plaques with telangiectasia and central atrophy on the lower leg

A 68‐year‐old woman presented to our dermatology clinic with erythematous plaques on her right lower limb in 2019. The patient was a known case of systemic sarcoidosis for 10 years. She was referred to our clinic with a 2‐month history of two yellow‐red plaques on her right lower limb, exactly located on the anterior part of the right thigh and right knee (Figure 1). She did not complain of pruritus, pain, or any other symptoms. She did not also report any similar cutaneous lesions over these years. The diagnosis of sarcoidosis had been made based on her lung involvement such as hilar lymphadenopathy and reduced pulmonary function tests that revealed reduced diffusion capacities and restrictive physiology. Then, she was on low dose oral steroid therapy. Moreover, her past medical history was not significant for any other disease.

In physical examination, there were nontender yellow‐red plaques with both telangiectasia and atrophy in the center. No other similar lesions were observed in other parts of her body.

**FIGURE 2:**
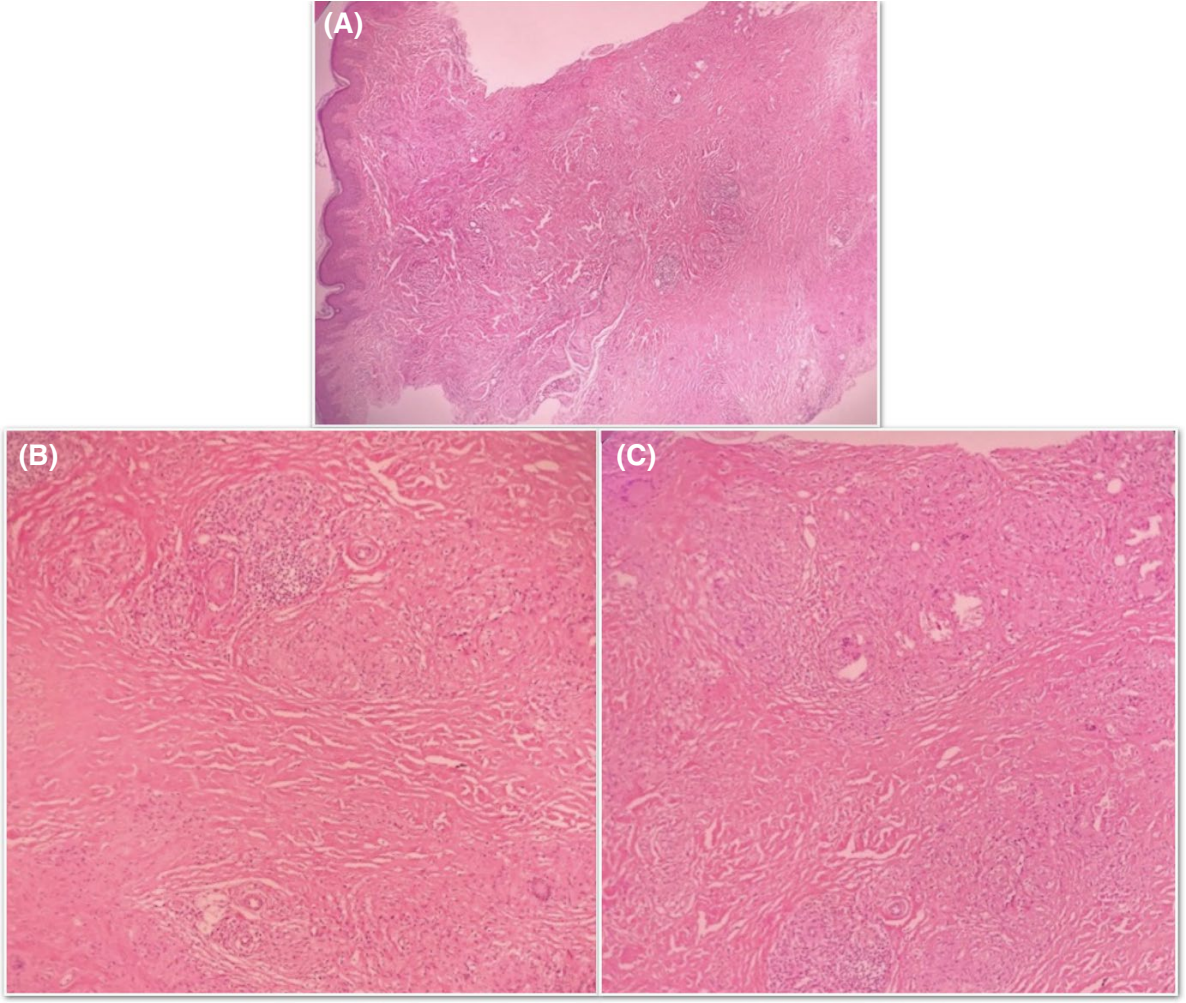
Histopathologic evaluation of the lesion: A, Zones of necrobiosis in dermis and subcutaneous tissue accompanied by dermal fibrosis and superficial and deep dermal perivascular inflammation, as well as few naked sarcoid‐type granulomas (×4 objective), B, Extensive necrobiosis surrounded by palisade of histiocytes and multinucleated giant cells (×10 objective), C, Some naked sarcoid‐type granulomas at the upper part of the image showing asteroid bodies (×10 objective)

The skin punch biopsy was taken from one of the lesions with the differential diagnosis of annular elastolytic giant cell granuloma, cutaneous sarcoidosis, granuloma annular, and necrobiosis lipoidica. Histological examination revealed several layers of necrobiosis in the dermis and subcutis surrounded by a palisade of histiocytes and giant cells, as well as dermal fibrosis and superficial and deep perivascular infiltration of lymphocytes and few plasma cells. Few scattered naked sarcoid‐type granulomas were also present. Furthermore, no dermal mucin deposition was seen in Alcian‐blue staining and no acid‐fast bacilli were seen in acid‐fast staining (Figure 2).

In her laboratory data, random blood sugar, fasting blood sugar, and hemoglobin A1C level (tested twice within 6 months period) all were in the normal range. She did not also mention any personal or family history of diabetes mellitus. Other laboratory data including serum calcium level, 25‐hydroxyvitamin D level, ESR were in the normal range. However, angiotensin‐converting enzyme (ACE) was above the normal range. Her clinical eye examination revealed no abnormality related to sarcoidosis, but her chest X‐ray demonstrated bilateral hilar lymphadenopathy.

The systemic treatment of sarcoidosis continued as before, with no alterations. Also, the topical steroid was prescribed for her skin lesions and no marked improvement was detected after several months.

## 3 DISCUSSION

Necrobiosis lipoidica diabeticorum was first described in 1929 as a cutaneous granulomatous lesion, sometimes associated with diabetes mellitus.^2^ Previous researches on NL described it as a consequence of diabetic microangiopathy, confirmed by Periodic acid‐Schiff staining and the presence of immunoglobulins in vessel walls of the lesions,^10^ although the majority of NL cases are not attributable to diabetes and thickening of vascular walls. It is thought that diabetes is detected in 65% of patient with NL lesions at the time of diagnosis while only 11% of diabetes develop NL in future.^1^

Herein, we report a rare case of systemic sarcoidosis that developed necrobiosis lipoidica‐like lesions in a nondiabetic patient. There are limited reports of NL development with concurrent sarcoidosis. We discuss 14 reported cases of this association in the literature ^1^, ^2^, ^3^, ^4^, ^5^, ^6^, ^7^, ^8^, ^9^, ^11^, ^12^ (Table 1). According to the previous studies, almost all the patients were female, aged 35 to 62, and had no past medical history of diabetes similar to our patient. The skin lesions were often located on the lower limbs and showed dermal epithelioid granulomas in histopathologic evaluations.^4^ However, it is not clear whether NL development with concurrent sarcoidosis might be coincidental or attributed to an association of these two diseases.

**TABLE 1:** Similar cases of concurrent systemic sarcoidosis and necrobiosis lipoidica‐like lesions and the associated features

Number	Sex/Age	Location of the lesion	Presence of necrobiosis	Presence of palisaded histiocytes	Presence of vascular changes	Presence of plasma cell	Presence of naked granuloma of sarcoidosis	Foamy histiocytes	Epidermal atrophy	Bx sites/Microscopic Dx	Time of the Dx of sarcoidosis/history of cutaneous sarcoidosis lesions	Year	Author
1	F/49 y	Shins	Yes	Yes	Yes	No	Yes	No	Yes	Shins: NL & S	6 y prior to NL	1957	Borire et al^11^
2	F/78 y	Scalp	No	Yes	No	No	No	Yes	No	Scalp: NL	9 y prior to NL	1976	Andersen et al^5^
3	F/35 y	Shins & arms	Yes	No	No	No	Yes	No	No	Shins: NL Arms: S	5 y prior to NL	1985	Graham et al^6^
4	F/42 y	Leg & cheeks	Yes	Yes	No	No	No	No	No	Legs: NL Cheeks: S	10 y prior to NL	1985	Graham et al^6^
5	F/61 y	Shins	Yes	Yes	No	No	No	No	No	Shins: NL	5 y prior to NL	1987	Monk et al^7^
6	F/48 y	Shin, arm, forehead, back	Yes	Yes	No	No	No	No	Yes	Shin: NL Arm, forehead,back: S	Simultaneously	1988	Dodd et al^8^
7	F/61 y	Shin & abdomen(scar)	Yes	Yes	No	No	No	No	No	Shin: NL Abdomen: S	Simultaneously	1991	Gudmundsen et al^9^
8	F/62 y	Shin and calf	No	No	No	No	Yes	No	No	Shin: S Calf: NL	10 y prior to NL	1998	Igawa et al^12^
9	F/53 y	Shin	Yes	Yes	No	Yes	No	Yes	No	Shin: NL	≥1 y prior to NL	2007	Mendoza et al^2^
10	F/38	Shin	Yes	Yes	No	Yes	No	Yes	No	Shin: NL	≥1 y prior to NL	2007	Mendoza et al^2^
11	F/54	Shin	Yes	Yes	No	Yes	No	Yes	No	Shin: NL	≥1 y prior to NL	2007	Mendoza et al^2^
12	F/64 y	Shins	Yes	Yes	No	Yes	Yes	No	No	S	6 y prior to NL	2012	Chiba et al^4^
13	F/58 y	Shins	Yes	Yes	No	Yes	No	No	No	NL	4 y after NL	2017	Valecha et al^1^
14	F/40 y	Shin	Yes	Yes	No	No	No	No	No	NL	3 y prior to NL	2018	Lee et al^3^

Note

Abbreviations: Bx, biopsy; Dx, diagnosis; NL, necrobiosis lipoidica; S, sarcoidosis; Y, year.

Cases are ordered regarding the year of publication.

Necrobiosis lipoidica usually presents as yellow‐brown to violaceous papules that develop into atrophic plaques with telangiectasia. Cutaneous sarcoidosis presents as red‐brown to violaceous papules or plaques, superficial nodules, or erythema nodusom. Various cutaneous manifestation of sarcoidosis is the possible explanation for describing it as the “great imitator” including even the atrophic telangiectatic plaques of NL, making its clinical diagnosis more difficult.^3^

Moreover, in microscopic examinations, collagen necrobiosis in lower dermis, foamy histiocytes, and lymphoplasmacytic infiltrations, all organized in horizontal layers, is characteristic of NL lesions while the presence of epithelioid‐cell granulomas is characteristic for sarcoidosis lesions.^4^ Therefore, confirming the microscopic diagnosis of sarcoidosis when collagen necrobiosis exists and discriminating it from NL, rheumatoid nodules, granuloma annulare, and infectious granuloma would be challenging.^13^

Thus far, different theories have been suggested to explain the coexistence of multiple cutaneous granulomatous diseases. In previous reports, sarcoidosis has been associated with other granulomatous diseases such as granuloma annulare, rheumatoid arthritis, and Crohn's disease. The possible explanation for these associations may be attributable to the similar cellular signaling mechanisms of granulomatous formation. In this regard, Macaron et al,^14^ indicated that granulomatous lesions expressed a considerable amount of glioma‐associated oncogene homolog‐1 (GLI‐1), which is a zinc finger transcription factor family. These factors are responsible for regulating the expression of the genes engaged in most of the essential cellular signaling processes, including embryonic development and cellular differentiation. It can be assumed that a shared mechanism of granuloma formation in different cutaneous granulomatous disorders can be responsible for their simultaneous appearance in a patient.^4^

Moreover, Ball et al^15^ suggested more various histologic changes in cutaneous sarcoidosis than was previously noted through discovering similarities between the histological features of systemic sarcoidosis and other granulomatous disorders, such as tuberculoid leprosy, rosacea, NL, granuloma annulare, and lichenoid inflammations caused by syphilis. Thus, this finding shows that NL‐like lesions, in a known case of systemic sarcoidosis, can be considered as a member of the broad spectrum of histologic changes in sarcoidosis. NL‐like lesions strongly resemble NL than typical cutaneous sarcoidosis as having more tendency to be ulcerated and resistant to systemic steroids as well. Therefore, further study is required to shed light on the etiology of coexistence of such lesions in some patients; however, it is necessary to rule out sarcoidosis in patients with NL lesions due to their possible association, especially in those without diabetes.

## CONFLICT OF INTEREST

None to declare.

## AUTHOR CONTRIBUTIONS

FA: involved in data gathering and preparing the manuscript. MT: involved in data gathering and preparing the manuscript.AR: involved in histopathology evaluation and preparing the manuscript. SD: involved in preparing the manuscript. RMR: involved in data gathering, supervising the whole report, and preparing the manuscript.

## ETHICAL APPROVAL

The ethical issues were completely considered to prepare this case report according to our institution ethical board guidelines. Moreover, this article was prepared regarding the declaration of Helsinki.
